# At least two well-spaced samples are needed to genotype a solid tumor

**DOI:** 10.1186/s12885-016-2202-8

**Published:** 2016-03-25

**Authors:** Kimberly Siegmund, Darryl Shibata

**Affiliations:** Department of Preventive Medicine, University of Southern California Keck School of Medicine, Los Angeles, CA USA; Department of Pathology, University of Southern California Keck School of Medicine, 1441 Eastlake Avenue , NOR2424, Los Angeles, CA 90033 USA

**Keywords:** Tumor heterogeneity, Mutation topography, Exome sequencing, Colorectal cancer

## Abstract

**Background:**

Human cancers are often sequenced to identify mutations. However, cancers are spatially heterogeneous populations with public mutations in all cells and private mutations in some cells. Without empiric knowledge of how mutations are distributed within a solid tumor it is uncertain whether single or multiple samples adequately sample its heterogeneity.

**Methods:**

Using a cohort of 12 human colorectal tumors with well-validated mutations, the abilities to correctly classify public and private mutations were tested (paired t-test) with one sample or two samples obtained from opposite tumor sides.

**Results:**

Two samples were significantly better than a single sample for correctly identifying public (99 % versus 97 %) and private mutations (85 % versus 46 %). Confounding single sample accuracy was that many private mutations appeared “clonal” in individual samples. Two samples detected the most frequent private mutations in 11 of the 12 tumors.

**Conclusions:**

Two spatially-separated samples efficiently distinguish public from private mutations because private mutations common in one specimen are usually less frequent or absent in another sample. The patch-like private mutation topography in most colorectal tumors inherently limits the information in single tumor samples. The correct identification of public and private mutations may aid efforts to target mutations present in all tumor cells.

**Electronic supplementary material:**

The online version of this article (doi:10.1186/s12885-016-2202-8) contains supplementary material, which is available to authorized users.

## Background

**Fig. 1:**
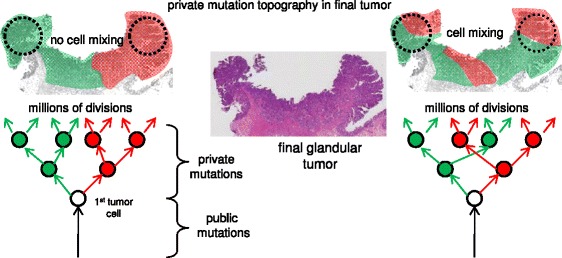
Colorectal tumors have glandular architectures (Cancer N is illustrated). Public and private mutations can be organized by ancestry, with private mutations acquired during growth. Depending on cell mobility, private mutations may segregate during growth into well defined “left” versus “right” patches, or more complex variegated patches. Importantly, a private mutation “clonal” in one bulk specimen (dotted circle) will usually be less frequent or absent in a sample taken from the opposite side

Current high-throughput DNA sequencers allow human tumor genotyping through targeted panels or with whole exomes or genomes [1]. Greater sequencing depths and better algorithms can more accurately measure mutations at increasingly lower frequencies. However, relatively unexplored is the optimal tumor sampling scheme. Multi-regional sampling of the same tumor illustrate that intratumoral heterogeneity (ITH), or different mutations in different cells, is very common in human tumors [2, 3]. Such ITH is not unexpected because mutations can arise during tumor growth (Fig. 1). Mutations can be divided into two groups based on when they were acquired during progression. Public (clonal) mutations are acquired before growth and are present in the first tumor cell and all its progeny. Private (subclonal) mutations acquired afterwards are present in only some tumor cells. For an exponential expansion, the frequency of a private mutation is lower the later it is acquired during growth.

For therapies directed against specific mutations, it is important to identify which mutations are present in nearly all cells. Therefore distinguishing public from private mutations is important. Various algorithms can infer whether a mutation is present in all cells (public) or in only some cells (private) from mutation frequencies and ploidy information (see for examples refs [4–6]). However, under certain scenarios, a private mutation may be frequent and therefore appear “clonal” in one portion of a tumor but be completely absent from another.

The crux of tumor sampling is whether the tumor cell population is uniform (well-mixed) or spatially heterogeneous. Liquid tumors such as leukemias are well-mixed but solid tumors such as colorectal adenocarcinomas (CRCs) have considerable physical structure (Fig. 1). In particular, colorectal adenomas and CRCs are composed of glands which partition cells into small discrete neighborhoods. Glands limit mixing and daughter cells would tend to remain adjacent. Moreover, during growth, cells with different private mutations could become widely separated in the final tumor, segregating private mutations into discrete subclonal patches (Fig. 1). Tumors with patch-like private mutation topographies would be impossible to characterize from single samples. The adequacy of a tumor genotype and optimal sampling schemes are uncertain without knowledge of tumor mutation topography. Here we demonstrate empirically with 12 human colorectal tumors (Table 1) that two widely-spaced samples provide significantly more information than single samples.

**Table 1:** Clinical data

Tumor	Type	Size (cm)	Stage	Glands examined	Data from ref 7
K	adenoma	6		6 left 6 right	yes
S	adenoma	6		4 left 4 right	yes
P	adenoma	3.5		3 left 4 right	yes
X	adenoma	2.5		5 left 5 right	yes
O	cancer	9.5	3	6 left 5 right	yes
A	cancer	5.6	1	4 left 5 right	new
C	cancer	6.4	3	5 left 5 right	new
M	cancer	3	2	7 left 7 right	yes
N	cancer	2.3	1	5 left 5 right	yes
W^a^	cancer	3.4	1	5 left 5 right	yes
U	cancer	3.9	2	5 left 5 right	yes
T	cancer	5.7	3	5 left 5 right	yes

^a^MSI+, rest are MSI-

## Methods

### Strategy

Tumor genotyping was previously reported for ten of the tumors [7, 8]. Briefly bulk samples (~0.5 cm^3^) were obtained from opposite tumor sides. Individual tumor glands were isolated with an EDTA washout, which yields nearly pure tumor cells free of normal stromal cells. Exome sequencing was performed on bulk DNA extracted from hundreds of glands, with mutations called with MuTect [9] at standard high confidence settings. Custom AmpliSeq panels (Thermo Fisher Scientific) were used to resequence the bulk specimens at selected loci, with an average depth of ~700X. Ploidy estimates at the loci were obtained with the OmniExpress SNP platform (Illumina). This study was approved by the ethics committee of the University of Southern California Health Sciences Campus.

Rigorously distinguishing between public and private mutations in human tumors is difficult and requires multiple samples. To define public and private mutations in these tumors, we also genotyped 7 to 14 individual tumor glands from the sides, because a mutation found on both tumor sides is not necessarily present in all cells. We defined public mutations as mutations present in both bulk samples and in all tumor glands. With the mutations rigorously defined, we can then test whether more limited sampling strategies (e.g. one bulk specimen) can reliably distinguish private from public mutations.

### Gland genotyping

Individual tumor glands contain ~10,000 adjacent cells. DNA was isolated using a crude lysis (TE and Proteinase K at 56 C for 4 h followed by boiling for 10 min [8]). The gland DNA (10 ng) was resequenced as with the bulk samples. Locus ploidy was estimated with high density SNP microarrays and pCBS [10] as with the bulk samples for 3 to 5 glands per side, using DNA extracted from the entire gland [7]. In general, ploidy at most chromosomal segments was identical between glands on a side, allowing this value to be applied to the resequenced glands. This ploidy information allows mutation frequency comparisons between public mutations (present in all tumors) and the private mutations. No correction for normal cell contamination was applied because the glands were nearly pure tumor cell populations.

### Tissue microdissections

Two other clinical specimens (paraffin blocks) were obtained from the tumors. Their spatial locations with respect to the bulk specimens are unknown. The topographical locations of selected public and private mutations were determined in approximately 8 to 18 small regions containing 3–5 glands microdissected [11] from their microscopic sections, followed by PCR and Sanger sequencing, with a manual call threshold of 5 % to call a mutation present. The numbers of mutations analyzed for each tumor are presented as Additional file 1.

### Driver mutations

Driver mutations were identified using the list proposed by Vogelstein et al. (Table S2A in ref [12]). Driver mutations were further evaluated by the mutationassessor.org website [13, 14], and had to be activating for oncogenes, or have medium to high impact or be a nonsense mutation for tumor suppressor loci.

### Statistics

A t-test (paired two sample for means) was used to compare the performances of one versus two samples for correctly calling public or private mutations.

## Results

### Public and private mutation frequencies often overlap in single samples

**Fig. 2:**
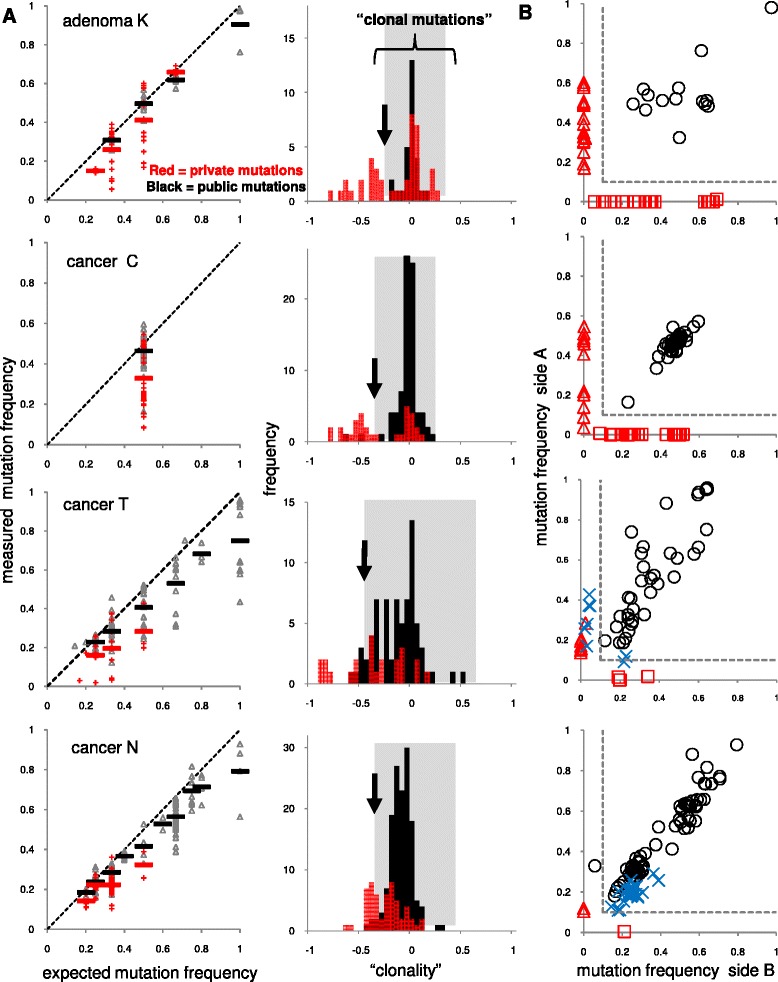
One versus two samples. **a** Mutation frequencies in single samples were plotted with respect to ploidy for public (black) or private (red) mutations for four representative tumors (see Additional file 2: Figure S1 for other tumors). Public mutations have a range of frequencies centered around their expected clonal values, which complicates classification because many private mutations also have frequencies that overlap with the public mutations. Black arrows indicate ad hoc cut points to distinguish public from private mutations. The grey shaded areas demonstrate that many private mutations have frequencies within the ranges of the public mutations, indicating that the private mutations are indistinguishable from the public mutations. Data from both single samples from the same tumor are presented. “Clonality” is calculated as: (measured mutation frequency - expected clonal frequency)/expected clonal frequency, with a zero value indicating the measured frequency is at its clonal value. **b** With two samples, public mutations are typically frequent on both sides. A private mutation frequent on one side is typically absent or rare on the other side. A simple 10/10 rule (<10 % frequency in one side, dotted lines) can usually accurately distinguish public from private mutations. A problematic case (Cancer N) illustrates that distinguishing public from private mutations in well-mixed cancers can be difficult, especially with aneuploid tumors. Blue X’s indicate private mutations found on both tumor sides

Mutation frequencies depend on tumor purity, locus ploidy, and whether the mutation is public or private. After correcting for ploidy and tumor purity, a mutation at a lower than expected clonal frequency may be a private mutation present in only some tumor cells. This type of analysis works best with high coverage (>100 X [4, 5]), with the coverage in this study ~700X. However, the validated public and private mutation frequencies were not distinct and often overlapped (Fig. 2a, with data from the 8 other tumors in Additional file 2: Figure S1). Public mutations have a spread of mutation frequencies around their expected clonal values, which reduces the precision of this approach. This variation likely reflects experimental confounders, including biases in the PCR and sequencing, which would require considerable effort to eliminate. At the same time, private mutations can also have mutation frequencies near their expected clonal values, resulting in their misclassification as public. This may occur if private mutations grow as well-defined subclonal patches in the final tumor (Fig. 1). Consequently, if a subclonal patch is sampled, its private mutations will be indistinguishable from its public mutations because both have clonal frequencies in that part of the tumor. Using ad hoc cut points to maximize the known classifications (Table 2), mutation frequencies usually identify public mutations (97 % average accuracy) but are relatively poor indicators of private mutations (46 % average accuracy) because many private mutations have “clonal” frequencies in the single specimens.

**Table 2:** One versus two tumor specimens

Tumor	Public mutations	Private mutations	Single biopsy	Single biopsy	Two biopsies	Two biopsies
			Fraction of public mutations correctly called	Fraction of private mutations correctly called	Fraction of public mutations correctly called	Fraction of private mutations correctly called
K	14	42	1	0.38	1	1
S	24	13	1	0.38	1	1
P	30	7	1	0.29	1	1
X	52	17	0.98	0.71	1	1
O	45	6	0.98	0.50	1	1
A	36	25	0.94	0.79	1	1
C	40	32	0.98	0.55	1	1
M	6	17	1	0.10	1	0.29
N	72	29	0.96	0.42	0.99	0.10
W	95	5	0.97	0.29	1	0.80
U	50	12	0.95	0.54	1	1
T	36	19	0.85	0.52	1	0.95
Average			0.97	0.46	0.99	0.85

### Two samples more accurately distinguishes public and private mutations

**Fig. 3:**
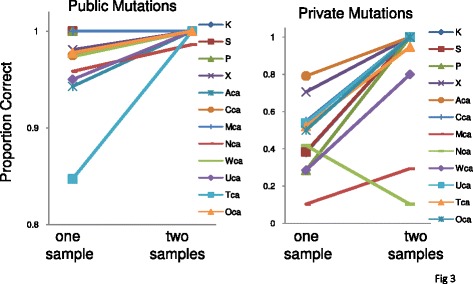
Two samples significantly improves the identification of most public and private mutations

In the absence of significant cell intermixing, a second sample can efficiently distinguish public from private mutations because a private mutation prevalent on one side of the tumor should be rare or absent on the opposite tumor side. A 10/10 rule was empirically employed to distinguish public from private mutations, with a private mutation having a frequency less than 10 % in one side (Fig. 2b). This two sample strategy was significantly better (Table 1) in identifying public mutations with an accuracy of 99.9 % (p = 0.026). It was also significantly better for identifying private mutations with an accuracy of 85 % (p < 5×10^−4^). Private mutation identification was improved for every tumor except one (Fig. 3). Reflecting tumor biology, less cell movement is expected in benign adenomas, and private mutations were completely side specific in the four adenomas. However, two of the 8 CRCs (Tumors M and N) were problematic because many of their private mutations were found at relatively high frequencies on both tumor sides, with correct assignment by the 10/10 rule for only 10 % and 29 % of the private mutations.

### Increased accuracy with topographical sampling

Another strategy to detect private mutations is to sequence smaller subpopulations such as single glands. Most tumor glands are clonal for both private and public mutations [7, 8] and therefore private mutations can be identified because they are absent from some glands. This single gland resequencing strategy was used to identify the public and private mutations in this study, but single glands are usually not available for analysis.

**Fig. 4:**
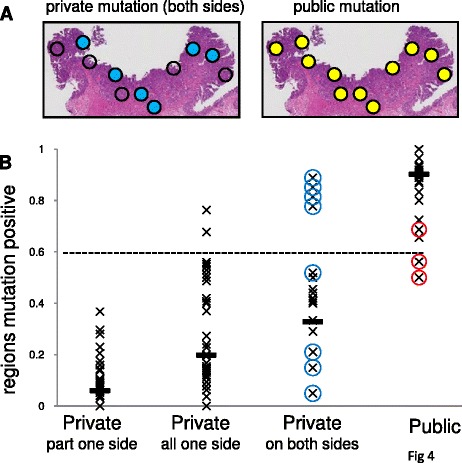
The topographical distributions of private mutations on microscope slides can also distinguish public from private mutations. **a** Public mutations are detected in most microdissected regions (yellow circles) but a private mutation found on both tumor sides is present in only some small regions (blue circles) in Cancer N. **b** Mutation topographic distributions on microscope slides can distinguish public from private mutations using a 60 % detection threshold (dotted line). Public mutations found in only some small areas (red circles) may be secondary to subsequent LOH. The high proportions of microdissected areas positive for the private mutations found on both sides in Cancers N and M (blue circles) may reflect that even immediately adjacent glands may have different private mutations in these well-mixed cancers

Instead, one can survey mutation topography in microscopic sections from readily available paraffin-embedded tissues (Fig. 4a). Multiple small tumor spots (3–5 glands) were microdissected from two different microscope slides for each tumor. A public mutation will be detected throughout the tumor whereas a private mutation will not. The efficiency of this method is somewhat diminished because some public mutations were detected in only some tumor regions, especially for loci that showed evidence of LOH (loss of multiple adjacent mutations) in the gland samples (Fig. 4b). LOH as a confounder of public mutations is further discussed in Additional file 1. Nevertheless, using a 60 % spot detection threshold, the method was 100 % accurate for private mutations present in only some glands on one side, 96 % accurate for private mutations that were “clonal” in one tumor side, and 74 % accurate for private mutations found on both tumor sides. Accuracy in calling public mutations was 94 %.

### When more than two samples are needed

**Fig. 5:**
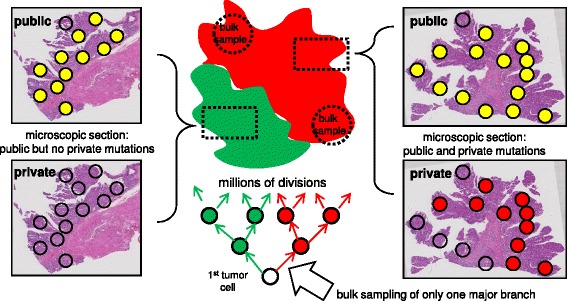
Hypothetical diagram illustrating how additional samples (microscopic sections, dotted boxes) can determine when two bulk samples (dotted circles) miss a major tumor tree branch. A public mutation (yellow circle) is present in nearly all the small regions but a private mutation (and 16 others) is missing from the left section of Cancer A. This finding suggests that the left tumor branch (green) was not sampled by the two initial bulk specimens

The topography of private mutations in the additional microscopic sections can also indicate when two bulk specimens do not adequately sample major tumor tree branches (Fig. 5). This shortcoming can be inferred if private mutations are completely absent from large regions of the microscopic tissue sections, indicating some early tumor branches were missed by the two bulk exome sequencing samples. This undersampling was present in one of the 12 tumors, where public but not private mutations were detected in one slide (Fig. 5). However, for the 11 other tumors, at least some of the private mutations detected in the bulk samples were also detected in the microscope sections, indicating the major branches of these tumor trees were likely sampled.

### Most driver mutations are public mutations

Generally driver and passenger mutations respectively segregated with public and private mutations (Table 3). However 3 of the 34 driver mutations (12 %) were private mutations not present in all tumor cells, indicating the potential for improper therapeutic targeting. Every tumor had at least one public driver mutation.

**Table 3:** Most driver mutations are public mutations

Tumor	Public (drivers)	Private (drivers)
K	14 (1)	42 (0)
S	24 (3)	13 (0)
P	30 (4)	7 (0)
X	52 (3)	17 (0)
O	45 (1)	6 (0)
A	36 (2)	25 (1)
C	40 (1)	32 (0)
M	6 (1)	17 (2)
N	38 (3)	17 (1)
W	95 (5)	5 (0)
U	50 (4)	12 (0)
T	36 (2)	19 (0)

## Discussion

Distinguishing public from private mutations is important for understanding tumor biology and for designing targeted therapies. Therapies against private mutations are unlikely to eliminate the tumor whereas public driver mutations are likely essential for tumorigenesis. ITH is common in human tumors, which complicates genotyping because mutations and their frequencies may differ throughout the tumor. Here we illustrate with 12 tumors the magnitude of the problem. It is difficult to distinguish private from public mutations in single samples because their mutation frequencies often overlap even when corrected for ploidy. Mutation frequencies provide no clear guide to public versus private mutations. By contrast, two samples from opposite tumor sides and a simple 10/10 rule more effectively identifies private mutations, even without ploidy information.

The efficiency of spatial sampling reflects that during growth, private mutations can only spread to parts of a tumor (Fig. 1). A subclonal mutation prevalent in one part of a tumor is by definition less common or absent in another part of the tumor. This spatial strategy becomes limited in well-mixed tumors, where private mutations are more evenly spread. This problem was observed in only 2 of the tumors, indicating that most colorectal tumors have well-defined patch-like private mutation distributions. Sequencing smaller tumor subpopulations (single glands or small regions on microscope slides) can further distinguish private from public mutations.

The “genotype” of a tumor is nebulous because tumors are populations of cells, and each cell is likely to have different mutations, as exemplified by single cell sequencing studies [15]. One systematic way to organize a tumor genotype is through ancestry, with public mutations present in the first tumor cell and private mutations acquired along the branches (Fig. 1). Because earlier mutations are more prevalent in growing populations [16], the major early tree branches are relatively easier to detect with current exome sequencing (about 10 % sensitivity [9]). Most primary colorectal tumors have simple star-like trees, reflecting single “Big Bang” expansions where most detectable private mutations arise early during tumorigenesis [7]. Consistent with the idea that private mutation frequencies depend primarily on when they occur during growth and not on selection, most private mutations appeared to be passive passengers acquired during the growth conferred by the public driver mutations.

Although spatial sampling requires sequencing three (“right” and “left” tumor and normal) rather than two samples, no ploidy information is required to classify public and private mutations. The patch-like topographies of subclones and their private mutations in many human colorectal tumors inherently limit the amounts of representative information that can be obtained from single tumor samples, whether for DNA sequencing or other biomarker measurements. Additional sampling and sequencing to greater depths will inevitable detect more private mutations, but in most cases, two widely spaced tumor samples appear to adequately sample the major tumor tree branches and their private mutations. Spatial sampling may be less effective in other solid tumor types where less glandular structure is present and cell mixing more extensive. Although tumor sequencing data are complex, simple tumor ancestral trees outline how and why spatial sampling is efficient.

## Conclusions

The empirical data in this study illustrate that two samples are significantly more accurate than a single sample for distinguishing public from private mutations in colorectal tumors. The correct identification of public mutations may aid efforts to target mutations present in all tumor cells.

## Additional files

Additional file 1:
**SOM: LOH can confound mutation classification.** (DOCX 423 kb) Additional file 2: Figure S1. Data from the 8 tumors not in Fig. 2. (PDF 223 kb)
