# Supplementary material for: At least two well-spaced samples are needed to genotype a solid tumor
Source: BMC Cancer. 2016 Mar 25;16:250. doi: 10.1186/s12885-016-2202-8 (PMC4807557; doi:10.1186/s12885-016-2202-8)
Supplement: Additional file 1: — SOM: LOH can confound mutation classification. (DOCX 423 kb) [file 12885_2016_2202_MOESM1_ESM.docx]

**SOM: LOH can confound mutation classification.** A public mutation is present in all tumor cells and acquired before the final expansion. However, a public mutation can falsely appear to be a private mutation if chromosomal instability during growth leads to the loss of a public mutation through LOH. The three public mutations in Fig 4 with the lowest frequencies of mutation positive microdissected spots were from a single tumor (“N”) and were not in all of the glands, initially indicating that they were private mutations. However, a tumor gland ancestral tree of the private mutations (as outlined in Fig 1) required these three mutations be acquired independently more than once. We noticed that the three mutations were present on the same chromosome segment, and therefore feel that a more likely scenario is LOH due to chromosomal instability during growth because LOH can simultaneously lead to the loss of multiple mutations. LOH is one of many potential pitfalls of distinguishing public from private mutations. Two samples from the same tumor provide additional information to help subclassify when mutations are acquired.

The numbers of mutations analyzed for each of the tumors by microdissection, PCR, and Sanger sequencing are present below.
