# Supplementary figures and images for: At least two well-spaced samples are needed to genotype a solid tumor
Source: BMC Cancer. 2016 Mar 25;16:250. doi: 10.1186/s12885-016-2202-8 (PMC4807557; doi:10.1186/s12885-016-2202-8)

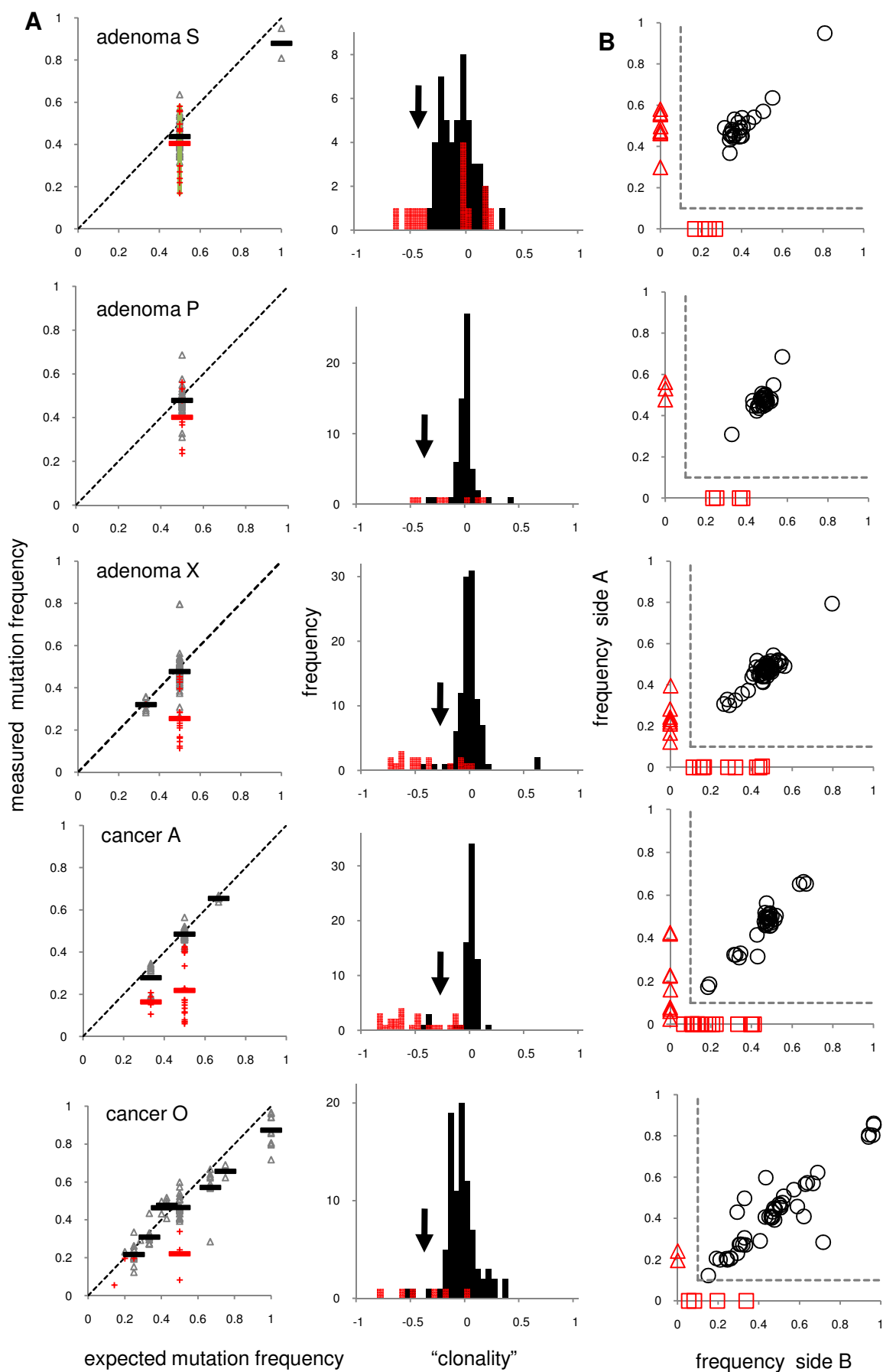

Supplemental Fig 1

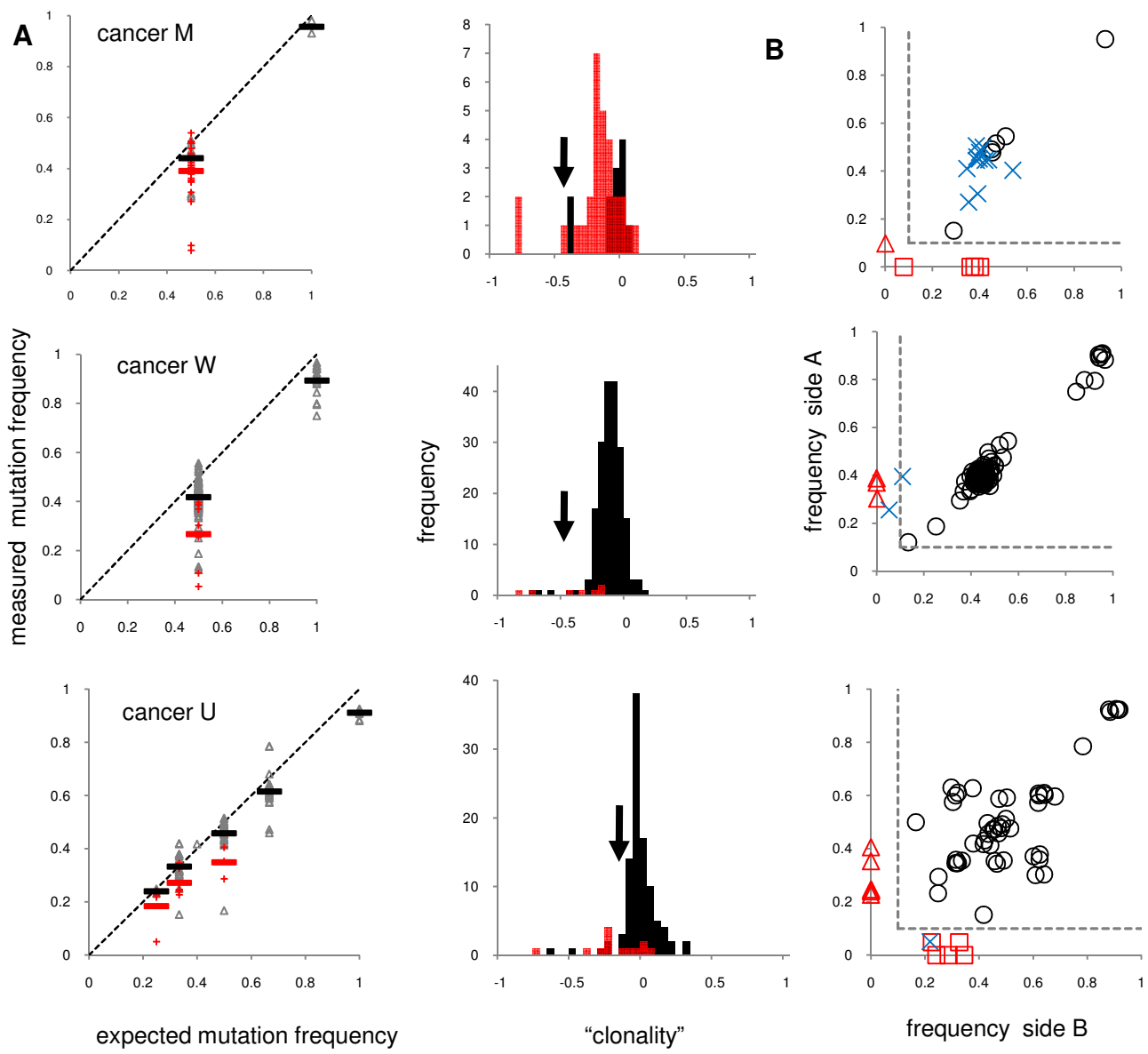

Supplemental Fig 1

Supplement: Additional file 2: Figure S1. — Data from the 8 tumors not in Fig. 2. (PDF 223 kb) [file 12885_2016_2202_MOESM2_ESM.pdf]
